# Sea anemones (Cnidaria, Actiniaria) of Singapore: redescription and taxonomy of *Phymanthuspinnulatus* Martens in Klunzinger, 1877

**DOI:** 10.3897/zookeys.840.31390

**Published:** 2019-04-17

**Authors:** Nicholas Wei Liang Yap, Ria Tan, Clara Lei Xin Yong, Koh Siang Tan, Danwei Huang

**Affiliations:** 1 Reef Ecology Laboratory, Department of Biological Sciences, National University of Singapore, Block S3 Level 4, Science Drive 4, Republic of Singapore; 2 St. John’s Island National Marine Laboratory, Tropical Marine Science Institute, National University of Singapore, 18 Kent Ridge Road, Republic of Singapore; 3 c/o Lee Kong Chian Natural History Museum, National University of Singapore, 2 Conservatory Drive, Republic of Singapore

**Keywords:** Actinoidea, Indo-Pacific, intertidal, Southeast Asia, zooxanthellae

## Abstract

Despite the ubiquity of sea anemones (Cnidaria: Actiniaria) in tropical ecosystems, our understanding of their biodiversity and taxonomy is limited. Here we re-establish the identity of an intertidal zooxanthellate species, *Phymanthuspinnulatus* Martens in Klunzinger, 1877. Originally described from a single preserved specimen in the Berlin Museum by CB Klunzinger, his brief footnote lacked crucial details to positively identify the species. Our redescription is based on more than 50 living individuals of *P.pinnulatus* collected from its type locality, Singapore. These were examined and compared with type materials of the species and its congeners. Specimens of *P.pinnulatus* differ from syntypes of species described as *Phymanthuslevis* Kwietniewski, 1898 from Indonesia, as well as *Phymanthussansibaricus* Carlgren, 1900 and *Phymanthusstrandesi* Carlgren, 1900, both described from East Africa. *Phymanthuspinnulatus* was encountered on the lower intertidal, among coral rubble and between rocky crevices. It is vibrantly coloured and has 96 marginal tentacles with branching outgrowths along each, resulting in a ‘frilly’ appearance. The anemone has a flat expanded oral disc, with discal tentacles that are inconspicuous and reduced, unlike syntypes of its congeners. Details of its live appearance, musculature, and cnidom are also provided for the first time. Overall, types of cnidae and capsule sizes differ from other known species of *Phymanthus* documented elsewhere. It is inferred that *P.pinnulatus* has a wide distribution that extends eastwards from Singapore, as far as Ambon and the Torres Straits. Some individuals reported as *Phymanthusmuscosus* Haddon and Shackleton, 1893 and *Phymanthusbuitendijki* Pax, 1924 are probably *P.pinnulatus*. This morphological analysis provides new insights into the characters used to delimit *P.pinnulatus*, clarifies its geographical distribution, and contributes to an ongoing revision of the genus *Phymanthus*.

## Introduction

Sea anemones (Cnidaria, Actiniaria) are ecologically successful invertebrates found in many tropical marine ecosystems. In spite of their ubiquity, few from the Indo-Pacific region have been subjected to rigorous taxonomic studies, and the identities of many species remain poorly defined (den Hartog 1997; Fautin et al. 2009). Among them are members of the zooxanthellate family Phymanthidae, which comprise two genera: *Heteranthus* and *Phymanthus* (Fautin 2013; 2016). Within the latter genus, *Phymanthuspinnulatus* [= *Phymanthuspinnulatum*] Martens in Klunzinger, 1877, was first described based on a single preserved specimen collected by Eduard von Martens from Singapore (Haddon 1898), and housed in the Berlin Museum (now Museum für Naturkunde). Its appearance was briefly described in a footnote by Klunzinger (1877: 87) stating “… wo statt der Wärzchen beim Lebenden (nach der Zeichnung von Martens) deutliche und mehrfach gefiederte Seitenstäbchen am Hauptstamm sitzen,” alluding to the presence of suckers [=verrucae] along the animal’s body, and ramified tentacles. Klunzinger’s (1877) footnote also makes mention to a drawing of the anemone by Martens. However, we were not able to locate it, nor does it appear in Martens’ comprehensive reports of biodiversity from his expedition in Southeast Asia (see Martens 1867, 1875).

Klunzinger’s footnote (1877) provided no further details or illustrations to support his description. Since then, the taxonomic validity of *P.pinnulatus*’ appearance has remained equivocal, with no illustrations or taxonomic work published thereafter to ascertain the identity of the species. Here we provide for the first time in over a century since Klunzinger’s (1877) description, details of *P.pinnulatus*’ external and internal structure (i.e., retractor and sphincter musculature), an inventory of cnidae [= cnidom], and notes on its habitat and distribution. We also provide field photographs of the living animal. These data are now convention in contemporary actinian taxonomic accounts.

We encountered *P.pinnulatus* at the lower intertidal zone, where its lower column was buried in sand or wedged between crevices of silt covered rocks and/or coral rubble. These anemones were very conspicuous and common in the northern and southern shores of Singapore. They were also easily recognizable in the field because of the frilly and colourful appearance of its marginal tentacles.

Prior to this study, 25 anemones which are well known taxonomically were identified from Singapore (see Fautin et al. 2009, 2015; Yap et al. 2014; Fautin and Tan 2016). Unlike these species, disagreements still persist for the diagnosis of *Phymanthus* and species attributed to this genus (Pax 1924; Carlgren 1949; Gonzalez-Muñoz et al. 2015), so clearly a taxonomical revision is overdue. Many members of *Phymanthus* are poorly defined. The objective of this study is to provide a comprehensive morphological characterization of *P.pinnulatus* so that its identity would be unambiguous. For a common tropical sea anemone, data are lacking on much of its biology, ecology, and biogeography. Our redescription opens up opportunities for further research on this common intertidal species in a wide range of disciplines.

## Materials and methods

All anemones we report here were collected from Singapore over 16 years: between 2002 and 2018. Some animals were observed *in situ* and photographed; others were brought back to the laboratory for further study. Collected anemones were kept alive for at least one week. Details on their behaviour and appearance of the living animal were noted. Thereafter, the whole animal was fixed in 10% formalin. Internal morphology was examined in dissected specimens. Musculature of the anemones was visualized from 8-µm-thick histological sections stained with haematoxylin and eosin (Humason 1967).

Unfired cnidae capsules were examined from tissues of the marginal tentacle tip, protuberances, discal tentacles, marginal projections, mid-column, actinopharynx, and mesenterial filaments. Cnidae were viewed at 1000 × magnification. We also examined discharged capsules from living specimens, to confirm identities of cnidae encountered (see Yap et al. 2014). Cnidae taxonomy follows Mariscal (1974).

We examined the holotype of *P.pinnulatum*, kept as two separate lots—one at the Museum für Naturkunde Berlin (ZMB) and the other at Naturhistoriska Riksmuseet, Stockholm, Sweden (NRS) (see Fautin 2016). Voucher specimens of individuals collected from Singapore by KW England and FB Steiner between 1960s and 1980s, deposited at Natural History Museum (known also as the British Museum of Natural History; BMNH) and California Academy of Sciences, Department of Invertebrate Zoology and Geology (CASIZ) respectively were also studied.

To further establish the identity of *P.pinnulatus* and to delineate the species, available and accessible type material of its congeners were examined: *Phymanthusbuitendijki* Pax, 1924 present at the Rijksmuseum van Natuurlijke Historie and Naturalis (RMNH; now the Naturalis Biodiversity Center); *Phymanthuslevis* Kwietniewski, 1898, present at both ZMB and NRS; *Phymanthusmuscosus* Haddon & Shackleton 1893, kept at the University Museum of Zoology, Cambridge University, United Kingdom (MZC); *Phymanthussansibaricus* Carlgren, 1900 and *Phymanthusstrandesi* Carlgren, 1900, both in the invertebrate collection at Zoologisches Museum Hamburg (ZMH).

We relied on published descriptions of *Phymanthuscrucifer* (Le Sueur, 1817), *Phymanthusloligo* (Hemprich & Ehrenberg in Ehrenberg, 1834), *Phymanthuspulcher* (Andrès, 1883) and *Phymanthusrhizophorae* (Mitchell, 1890) to obtain details of their appearance and morphological traits. The types of *P.pulcher* and *P.rhizophorae* could not be located (Fautin 2013, 2016).

While syntypes of both *P.crucifer* and *P.loligo* do exist and are present at the Museum of Zoology, Lund University (MZL), these are in the form of microscope slides of the anemones’ mesenteries and musculature. The slides alone are not useful for defining species boundaries of members in *Phymanthus*. Furthermore, we did not study the syntype of *Phymanthuscoeruleus* (Quoy & Gaimard, 1833) because specimens present in the lot are of two different anemone species (Fautin 2013).

Because uncertainty still lies with distinguishing the two genera of Phymanthidae (i.e., *Phymanthus* and *Heteranthus*; see González-Muñoz et al. 2015), to verify that *P.pinnulatus* is morphologically distinct from members of *Heteranthus*, we examined the syntype of *Heteranthusverruculatus* Klunzinger, 1877 and holotype of *Heteranthusinsignis* Carlgren, 1943, kept also at Museum für Naturkunde Berlin (ZMB) and Naturhistoriska Riksmuseet, Stockholm, Sweden (NRS) respectively.

All new *P.pinnulatus* voucher specimens collected from Singapore for this study since 2002 were deposited in the Zoological Reference Collection, Lee Kong Chian Natural History Museum, National University of Singapore (**ZRC**).

## Taxonomic Account

### Family Phymanthidae Andres, 1883

#### Genus *Phymanthus* Milne-Edwards, 1857

##### Phymanthus pinnulatus

Taxon classificationAnimaliaActiniariaPhymanthidae

Martens in Klunzinger, 1877

Figs 2 , 3 , 4 , 5 , 6 , 7

Phymanthus pinnulatum Martens in Klunzinger, 1877: 87 (original description).
Phymanthus pinnulatum : Haddon 1898: 496; Carlgren 1949: 75.
Phymanthus pinnulatus : Fautin 2016: 346.

###### Occurrence and materials collected in Singapore

**Figure 1.**
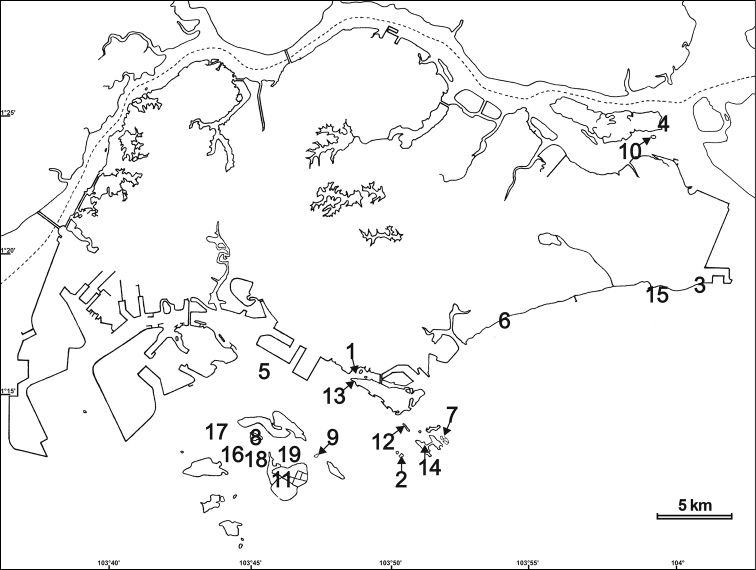
Map of Singapore where specimens of *Phymanthuspinnulatus* were collected for this study: 1, Berlayer Creek (1°15'56"N; 103°48'25"E); 2, Big Sisters’ Island (Pulau Subar Laut) (1°12'50"N; 103°50'05"E); 3, Changi East Beaches (1°18'45"N; 104°00'31"E); 4, Chek Jawa (1°24'25"N; 103°59'23"E); 5, Cyrene Reef (Terumbu Pandan) (1°15'28"N; 103°45'19"E); 6, East Coast Park Beaches (1°17'36"N; 103°53'46"E); 7, Kusu Island (Pulau Tembakul) (1°13'25"N; 103°51'39"E); 8, Pulau Hantu (1°13'35"N; 103°45'03"E); 9, Pulau Jong (1°12'54"N; 103°47'12"E); 10, Pulau Sekudu (1°24'19"N; 103°59'17"E); 11, Pulau Semakau (1°11'58"N; 103°45'31"E); 12, Pulau Tekukor (1°13'51"N; 103°50'18"E); 13, Sentosa (Tanjong Rimau) (1°14'47"N; 103°49'56"E); 14, St John’s Island (1°13'17"N; 103°50'55"E); 15, Tanah Merah (1°18'45"N; 103°59'34"E); 16, Terumbu Bemban (1°12'36"N; 103°44'27"E); 17, Terumbu Pempang Tengah (1°13'33"N; 103°43'50"E); 18, Terumbu Raya (1°12'46"N; 103°45'09"E); 19, Terumbu Semakau (1°12'46"N; 103°46'07"E).

(Fig. 1). (* – observed alive; **bold** – morphotypes with smooth marginal tentacles or reduced protuberances):

Berlayer Creek (ZRC.CNI.1343 x4*), Big Sister’s Island (ZRC.CNI.0982 x1; ZRC.CNI.1103 x1*; ZRC.CNI.1163 x1*; ZRC.CNI.1045 x1*; ZRC.CNI.1347 x4*), Changi East Beaches (ZRC.CNI.1084 x1*; ZRC.CNI.1106 x1*), Chek Jawa (photographed but not collected), Cyrene Reef (ZRC.CNI.1089 x1*; ZRC.CNI.1112 x2*; ZRC.CNI.1145 x1*; ZRC.CNI.1342 x4*), East Coast Park Beaches (ZRC.CNI.1039 x1*; ZRC.CNI.1046 x1*; ZRC.CNI.1110 x1*), Kusu Island (ZRC.CNI.1162 x1*), Pulau Hantu (ZRC.CNI.0015 x1; BMNH1995.1006 x1; CASIZ161242 x1), Pulau Jong (**BMNH1996.355 x1**), Pulau Sekudu (ZRC.CNI.0738 x1), Pulau Semakau (ZRC.CNI.1031 x1*; ZRC.CNI.0318 x1; ZRC.CNI.0321 x1; **ZRC.CNI.0322 x1**; ZRC.CNI.0639 x1; ZRC.CNI.1098 x1*; ZRC.CNI.1361 x1*), Pulau Tekukor (ZRC.CNI.0993 x3*, of these only one has reduced protuberances; BMNH1996.313 x1; ZRC.CNI.1306 x1*), Sentosa (Tanjong Rimau) (ZRC.CNI.1345 x4*), St John’s Island (ZRC.CNI.0467 x1), Tanah Merah (photographed but not collected), Terumbu Bemban (ZRC.CNI.1223 x1*), Terumbu Pempang Tengah (ZRC.CNI.1028 x1*; ZRC.CNI.1029 x1*), Terumbu Raya (ZRC.CNI.1111 x1*), Terumbu Semakau (ZRC.CNI.0493 x1).

###### Type material examined.

**Figure 2.**
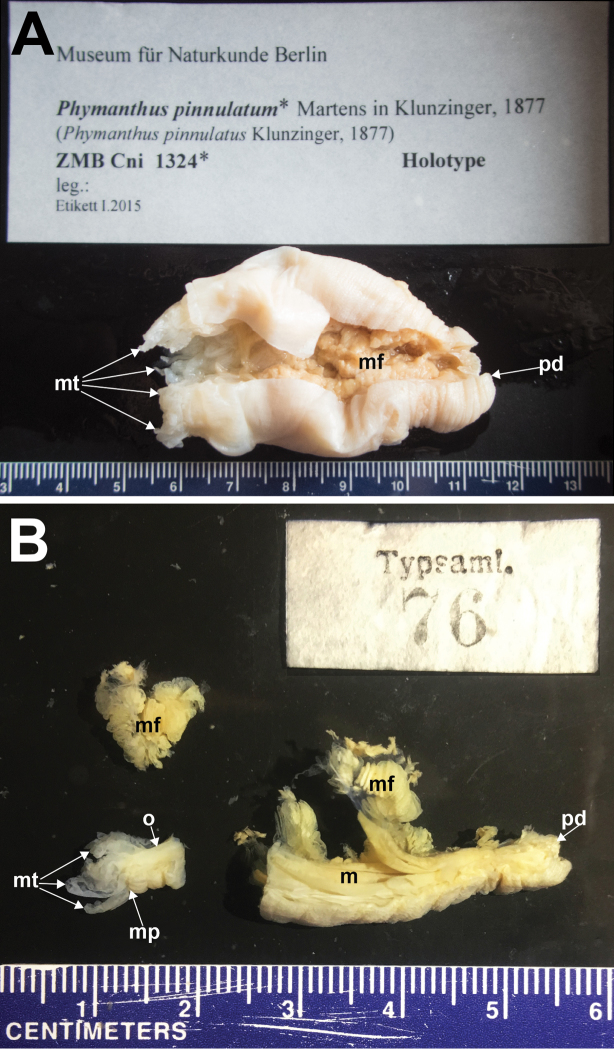
Holotype of *Phymanthuspinnulatum* Martens in Klunzinger, 1877 **A** entire specimen present at the Museum für Naturkunde, Berlin (ZMB Cni 1324), Germany **B** three pieces of the holotype removed from the Berlin specimen now at Naturhistoriska Riksmuseet (NRS76), Stockholm, Sweden. Abbreviations: m, mesenteries. mt, marginal tentacles. mf, mesenterial filaments. mp, marginal projection. o, oral disc. pd, pedal disc. Photographs by NWL Yap.

Holotype, ZNB Cni 1324, collected by E. von Martens. A single specimen, 60 mm in length, flaccid, cut longitudinally, a slice of the distalmost margin and part of the proximal end missing, though a little of the pedal disc remains, cream-coloured entirely (Fig. 2A); NRS76 consists of three pieces originating from ZMB Cni 1324 (see Fautin 2016), all pieces cream-coloured in preservative: a piece of the distalmost end with oral disc and marginal tentacles present, 11 mm in length; a piece of mesentery, fertile, 9 mm wide; a 30 mm longitudinal strip of the column (Fig. 2B).

###### Natural history.

**Figure 3.**
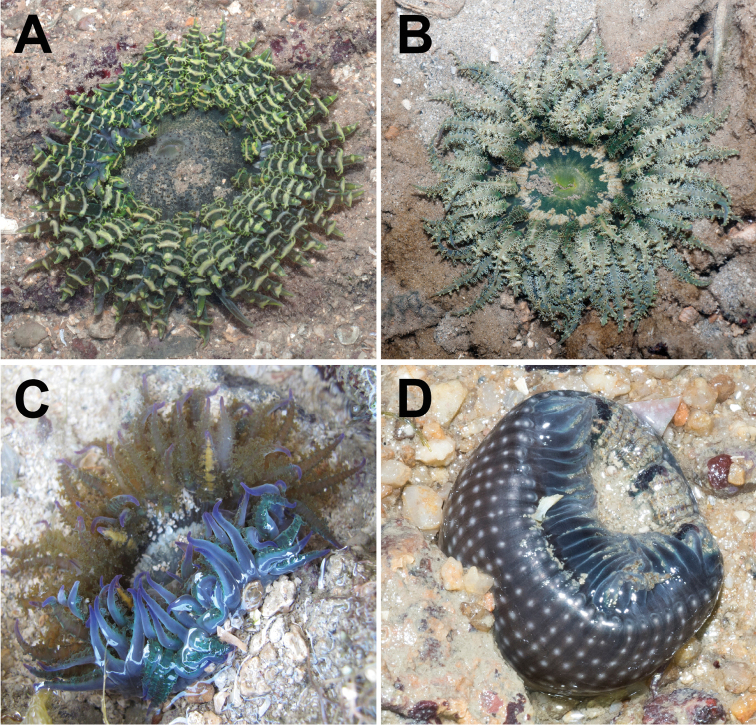
Living specimens of *Phymanthuspinnulatus*, external morphology of oral end **A** expanded individual of green “banded” colour morph, in situ. Photograph by R Tan **B** an expanded slaty-green “plain” coloured individual, with extensive branching of its protuberances, in situ. Photograph by R Tan **C** a third colour morph with brilliant electric blue marginal tentacles, in situ. Photograph by NWL Yap **D** a partially contracted individual in situ, with its oral end protruding from the substratum; note longitudinal rows of verrucae along intermesenterial spaces, extending proximally from the oral end towards mid column, in situ. Photograph by R Tan.

Usually encountered during low tide, with upper portion exposed, oral disc and marginal tentacles expanded (Fig. 3A, B, C). Sediment and small shell fragments may adhere to verrucae (Fig. 3D). Lower body usually deep in crevices or buried in sand or coral rubble. Pedal disc attached to buried rock, fragments of shell or coral rubble. Retracts quickly and deeply into substratum when disturbed, pulling in marginal tentacles completely. Animal typically found singly, with multiple individuals separated by a short distance (> 20 cm), although clusters up to four have been observed. Zooxanthellate.

###### Marginal tentacles.

**Figure 4.**
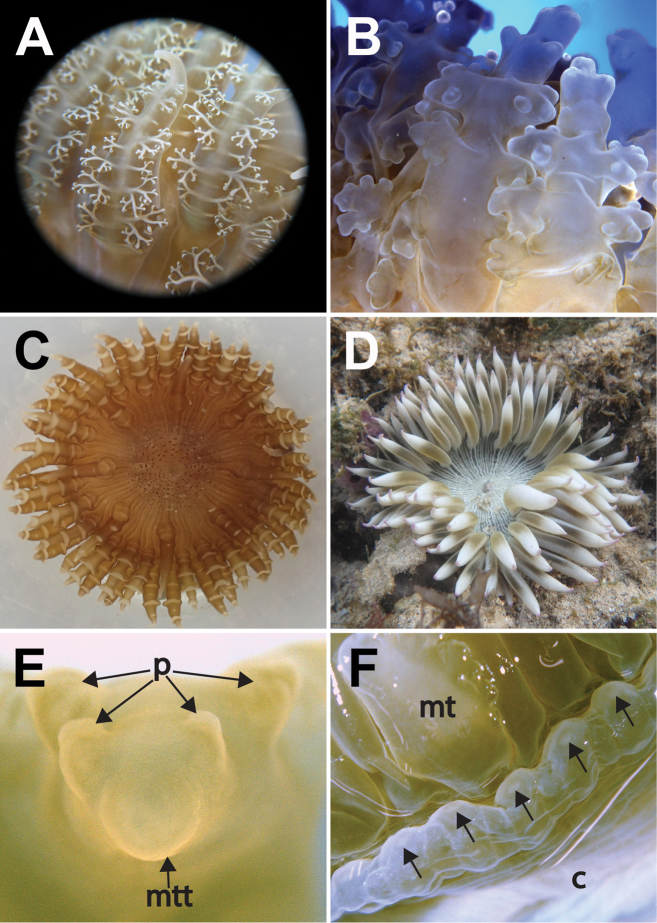
Marginal tentacle and marginal projection appearance of *Phymanthuspinnulatus***A** close-up of ramified protuberances of a living specimen. Photograph by NWL Yap **B** close-up of protuberances from a fixed specimen (ZRC.CNI.1345); note that finer details of protuberance branching are lost in preserved/fixed specimens, branching of protuberances now appear as knobs. Photograph by NWL Yap **C** a morphotype of that lacks ramified protuberances. Note that this individual (ZRC.CNI.1029) is atypical as it has lesser marginal tentacles, by which are also octo-ramously arranged. Photograph by NWL Yap. **D** a “smooth” tentacle morphotype, in situ. Photograph by R Tan **E** close-up of a marginal tentacle tip from a fixed specimen (ZRC.CNI.1342). Note absence of perforation at tip **F** close-up of a row of marginal projections of a fixed specimen (ZRC.CNI.1342). Note perforations (arrowed). Abbreviations: mt, marginal tentacles; mtt, marginal tentacle tip; p, protuberances. Photographs by NWL Yap.

96 in total; one individual with 98 (ZRC.CNI.1342). All of similar length, equal to oral disc radius or longer (Figs 3A, B). Arranged hexamerously in four cycles but octamerously in one individual (ZRC.CNI.1029). Cycle closest to margin exocoelic; innermost cycles endocoelic. One per endo-/exocoel. Ramified protuberances occur laterally along both sides, symmetrical, alternating between large and small knobs (Fig. 4A). In life, branching appears extensive; when preserved, appears as low knobs (Figs 4A, B, respectively). Extent of branching may vary; some individuals have protuberances as slight bumps while in others the entire length is smooth (Figs 4C, D). Tip narrow and blunt, without perforation (Fig. 4E); base wide. Colour variable, from greenish-brown, slatey-grey to blue with golden tinge (Figs 3A, B, C); tip with green, purple, or pink cast. In fixed individuals, tentacles cream-coloured to greenish, translucent. Protuberances cream-coloured to gold on oral side; usually with a white line adjoining opposite protuberances (Fig. 4A).

###### Column.

Colour variable, from tan to translucent white. Distalmost end dark-brown. May appear whitish or cream-coloured in life, or with a light green tinge in preserved specimens. Distalmost end flared outwards when animal is expanded; mid-section uniform diameter; pedal end may spread outwards when animal is attached to a surface. Diameter of distalmost end greater than pedal disc. Marginal projections present along margin of distalmost end; may be inflated, perforated (Fig. 4F), with a central white dot. Dot not visible in preserved specimens. Longitudinal rows of adherent verrucae present, extending proximally to mid-section (Fig. 3D). In life, shell fragments or substratum particles may be attached to verrucae. Verrucae outline eye-shaped, as low white bumps, middle depressed, diameter <1 mm. Verrucae rows endocoelic; alternate long and short rows. Longer rows with typically more than eight verrucae; shorter rows with less than five. Mesenterial insertions seen as white lines that extend from distalmost to pedal end. Past mid-section: plain and smooth, without any obvious structures. Cinclides present, visible only when limbus is expanded. Fosse present, shallow, ca. 1 mm deep.

###### Oral disc.

**Figure 5.**
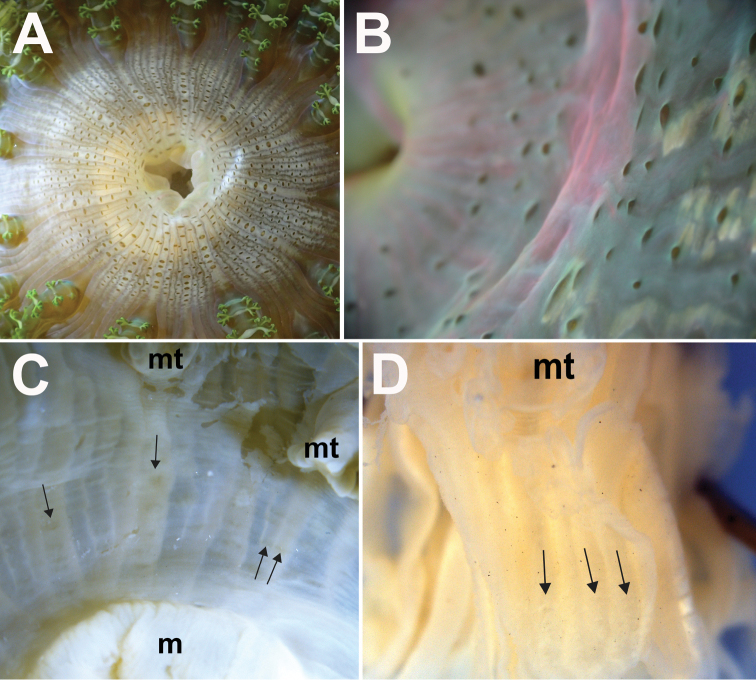
Detail of the oral discs of *Phymanthuspinnulatus*, external morphology **A** top view of discal tentacles present on a live individual, arranged as radial rows between intermesenterial spaces, extending from the mouth towards the region of the marginal tentacles (ZRC.CNI.1361) **B** side view of discal tentacles of a living specimen; note the low and reduced elevation of tentacles (ZRC.CNI.1046) **C** faint and reduced radial rows of discal tentacles (arrowed) present on a recent, formalin-fixed specimen (ZRC.CNI.1345) **D** very reduced and barely noticeable remnants of discal tentacles (arrowed) present on the holotype (NRS76); note that this specimen was preserved before 1877. Abbreviations: m, mesenteries; mt, marginal tentacles. Photographs by NWL Yap.

Outline round, flat when fully expanded; diameter 40 mm or greater. Colour in life grey to dark brown, with white markings flanking outwards; in fixed specimens, cream-coloured to translucent white. Discal tentacles present, arranged in radial rows extending from mouth to marginal tentacles, both endo- and exocoelic, numerous in a row (Fig. 5A). Discal tentacles outline: slim oval, as low bumps (Fig. 5B), some with middle sunken in, dependent on state of expansion. Discal tentacles dark-brown or grey in life. In preserved individuals, these are very inconspicuous, seen as horizontally radiating short grey dashes (Fig. 5C), may be very faint, or not seen at all, depending on state and age of specimen (e.g. Figs 5C, D). Wall thin; dark lines corresponding to mesenterial insertions visible through wall, extends from the mouth to margin. Central mouth oval and flat, area around it may be translucent. Two siphonoglyphs, symmetrical. In life, these may be white with pinkish streaks. Preserved, siphonoglyphs appear cream-coloured.

###### Pedal disc.

Oval, flat, same colour as proximal section of column. Thin-walled, mesenterial insertions appear as radiating white lines. Strongly adherent; readily attaches to surfaces to follow contour of substratum.

###### Internal morphology.

**Figure 6.**
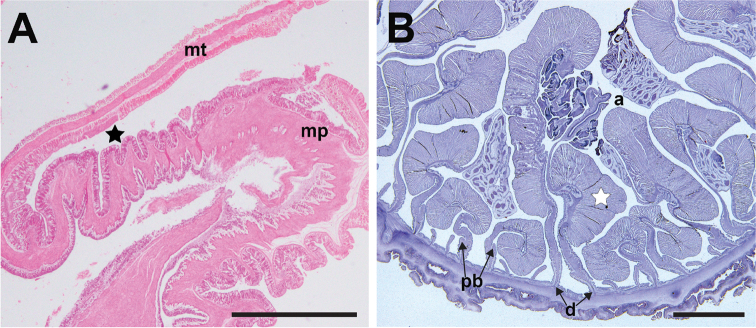
Internal musculature of *Phymanthuspinnulatus***A** longitudinal section of ZRC.CNI.0321 at the margin, showing lack of marginal sphincter muscle. Fosse is indicated by a black star **B** cross-section of ZRC.CNI.0993. Note the presence of well-developed retractor muscles (white star), and oocytes. Abbreviations: a, actinopharynx; d, directives; mp, marginal projection; mt, marginal tentacle; pb, parietolbasilar muscle. Scale bar: 10 mm. Photographs by NWL Yap.

Actinopharynx longitudinally pleated, extends proximally until mid-column. Oral and marginal stomata present. Mesenteries contain zooxanthellae, arranged in three orders. All 12 pairs of highest order complete, fertile and with filaments; two of these directives, each attached to a siphonoglyph. Mesenteries of second order incomplete, but all fertile with filaments, 12 pairs. In one individual (ZRC.CNI.0467) nine pairs of imperfect mesenteries were present in the second order. Twenty-four pairs of mesenteries small, without filaments and retractor pennon make up third order. All mesenteries, except smallest, extend to the proximal end. Sphincter muscle absent (Fig. 6A). Retractor muscles: strong, diffuse to diffuse circumscript. Parietobasilar muscle extends away from mesentery, as a reduced pennon (Fig. 6B), poorly developed. No internal broods encountered.

###### Cnidom.

**Figure 7.**
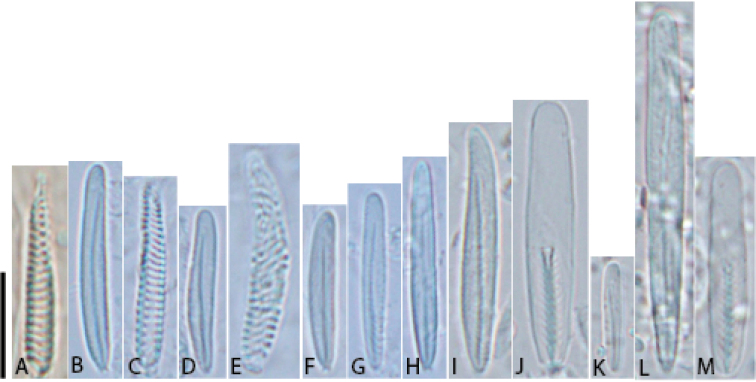
Cnidae of *Phymanthuspinnulatus***A** spirocyst **B** basitrich **C** spirocyst **D** basitrich **E** spirocyst **F** basitrich **G** basitrich **H** basitrich **I** basitrich **J** microbasic *p*-mastigophore **K** small basitrich **L** large basitrich **M** microbasic *p*-mastigophore. Scale bar: 10 μm. Photographs by NWL Yap.

Spirocysts, basitrichs, microbasic *p*-mastigophores (Table 1). Cnidae illustrated in Fig. 7. No cnidom data yielded from holotype (i.e., ZMB Cni 1324 and NRS 76), cnidae present damaged with crystalline appearance.

###### Distribution.

Singapore (Klunzinger 1877; this study), Indonesia (pers. obs.; see discussion below) and Northern Australia (see discussion below).

**Table 1.** Cnidom of *Phymanthuspinnulatus* Martens in Klunzinger, 1877. Measurements in μm; size outliers of single capsules are presented in values within parentheses. Abbreviations: N = the number of specimens having that type of cnidae to total specimens examined; n = total number of capsules measured for each type. Letters in parentheses following cnidae type refer to its illustration in Fig. 7.

**Tissue**	**Cnidae**	***Phymanthuspinnulatus* Martens in Klunzinger, 1877**		
		**Range length × range width**	**N**	**n**
Marginal tentacles	Spirocysts (A)	13.5–26.5(27.5) × 2.0–4.2	9/9	91
	Basitrichs (B)	14.7–25.7(26.5) × 2.0–3.7(4.7)	9/9	93
Protuberances	Spirocysts (C)	(10.0)13.0–24.0 × 2.0–4.0	9/9	90
	Basitrichs (D)	11.0–21.0 × 2.0–3.0	9/9	100
Discal tentacles	Spirocysts (E)	14.0–26.0 × 2.0–4.5(5.0)	9/9	90
	Basitrichs (F)	(8.0)10.0–19.0 × 2.0–3.0	9/9	90
Marginal pseudoacrorhagi	Basitrichs (G)	12.0–17.0 × 2.0–3.0	9/9	100
Column	Basitrichs (H)	13.0–23.1(25.0) × 2.0–3.7(4.2)	9/9	100
Actinopharynx	Basitrichs (I)	11.5–25.5 × 2.0–4.5	9/9	100
	Microbasic *p*-mastigophores (J)	14.7–25.7 × 4.0–7.9	9/9	88
Mesenterial filaments	Small basitrichs (K)	10.0–23.1 × 2.0–3.7	9/9	92
	Large basitrichs (L)	(26.3)27.0–38.0 × (2.6)3.0–4.7	9/9	91
	Microbasic *p*-mastigophores (M)	15.8–25.0 × (2.5)3.5–5.5(6.3)	9/9	92

###### Remarks.

Of the 53 specimens collected in this study and those examined in situ, we encountered five individuals having reduced protuberances. These morphotypes were only encountered along the southern Singapore shores.

While Klunzinger (1877) makes no mention of the etymology of the name, nor does it appear in Martens’ (1867, 1875) reports, the original species name is rendered as *pinnulatum*, made up of both a noun (*pinnula* = small wing) and a neuter, adjective forming suffix (-*tum*), thereby making it an adjective in the nominative singular (ICZN Article 11.9.1). Therein, the original spelling of the species name is incorrect. The species name, being an adjective in a genitive case, according to Article 31.2 of the International Code of Zoological Nomenclature (International Commission on Zoological Nomenclature 1999), must agree in gender with the genus. The gender of *Phymanthus* is masculine, therefore the species name is *pinnulatus* (see also Fautin 2016).

## Discussion

### Discal tentacles

A feature that unites pieces of holotype (i.e., ZMB Cni 1324 and NRS76), vouchers and fresh specimens of *Phymanthuspinnulatus* (N = 53), is the appearance of discal tentacles. For all individuals examined here, this feature is inconspicuous, reduced and oval; occurring as faint and dark horizontal dashes radiating outward from the mouth in preserved specimens (Figs 5A and C) and in older preserved specimens, less obvious (Figs 5D). There is no mention of discal tentacles whatsoever in Klunzinger’s (1877) description, and it is likely he described what was most obvious from the Berlin specimen. Regardless of anemone size or location where it was collected, shape and size of discal tentacles were consistent in its form for all materials we examined.

The inconspicuous and reduced form of discal tentacles in *P.pinnulatus* is distinct from its congeners depicted in primary scientific literature, and of type materials we studied. Those of *P.crucifer*, *P.loligo*, and *P.rhizophorae* are illustrated to be conspicuous and papilliform (see *P.crucifer*: Durden 1900: pl 10, fig 1; *P.loligo*: Klunzinger 1877: pl. 6, fig. 7, pl. 7, fig. 3 and Carlgren 1900: pl. 2, fig. 3; *P.rhizophorae*: Mitchell 1890, pl. 36, fig. 5). Discal tentacles present on syntypes of *P.sansibaricus* (ZMH C2620 and ZMH C2627) and *P.strandesi* (ZMH C2585) are also conspicuous and papilliform, like those depicted for *P.loligo*. Among syntypes of *P.levis* (ZMB.CNI.3811, NRS5557), we found that discal tentacles of this species are very different: they resemble marginal tentacles stunted in growth, unlike those of *P.pinnulatus* and *P.loligo*.

On *P.muscosus* found nearby (i.e., Indonesia and northern Australia), Kwietniewski (1898: 420) wrote “Sonst erscheint diese Partie der Mundscheibe ganz glatt, und nur nach sehr sorgfältiger Prüfung fand ich auf mehreren Sectoren der Mundscheibe Reihen von runden, äusserst geringen Erhebungen, welche als die Rudimente der scheibenständigen Tentakel zu deuten sind,” referring to slight bumps as ‘rudiments of disc-like tentacles,’ visible after ‘careful examination’, and an apparent overall smoothness to the area around the mouth. Moreover, illustrations in Kwietniewski’s report (1898: pl. 29, figs. 57, 58) show *P.muscosus* with a smooth oral disc, without discal tentacles. Similarly, Pax (1924) makes no mention of any discal tentacles found on the oral disc of *P.buitendijki*. Both Kwietniewski (1898) and Pax (1924), did not examine the holotype of *P.pinnulatus*; neither did workers before and after them (e.g. Haddon and Shackleton 1893; Haddon 1898; Stephenson 1922; Carlgren 1949, 1950). Given the close geographical proximities between these three reported species, it is possible that some individuals described by them as *P.muscosus* and *P.buitendijki* are in fact *P.pinnulatus* instead. Having examined the syntypes of *P.muscosus* collected by Dr AC Haddon, and *P.buitendijki* that Pax (1924) had examined, we found at least one resembling *P.pinnulatus* within the lots (i.e., MZC.I.33745 and RMNH.COEL.3876).

Discal tentacle form appeared to be consistent for all *P.pinnulatus* type and voucher materials examined here. We propose that this trait could be a stable character to help infer and define species boundaries for members of *Phymanthus*. As of writing, many members of this genus remain poorly described; whether the discal tentacle form is truly a useful trait to define species boundaries among members of *Phymanthus* warrants further study.

### Cnidom

In this study, we report upon the cnidom of *P.pinnulatus* (Table 1, Fig. 7) for the first time. Although cnidom data are a necessary component in modern actinian taxonomic descriptions (Fautin 1988), none have been reported in the original description. All three cnidae types (i.e., spirocysts, basitrichs and microbasic *p*-mastigophores) were found in tissues of *P.pinnulatus* examined for this study; these agree with the diagnosis of the genus *Phymanthus* (Carlgren 1949: 74). Because the use of cnidom data in anemone systematics only became routine after the 1940s (Fautin 1988), many published descriptions of other *Phymanthus* species we reviewed did not have any cnidae type or capsule size data for comparison (e.g. Haddon and Shackleton 1893; Kwietniewski 1898; Pax 1924) as these descriptions were published before the 1940s. Among reports that had cnidae size data, we found those of *P.crucifer* from the Gulf of Mexico (see Gonzalez-Muñoz et al. 2015), *P.pulcher* from the Aegean Sea (see Chintiroglou and den Hartog 1995) and *Phymanthusmuscosus* from the Great Barrier Reef (Carlgren 1940, 1950) to be sufficiently detailed and therefore useful for comparison. Cnidae sizes and types of *P.pinnulatus* were consistently different from *P.crucifer* and *P.pulcher*. Basitrichs in the mesenterial filaments of *P.pinnulatus* were much longer than those found in *P.crucifer* (basitrich length: *P.pinnulatus* = 27.0–38.0 μm; *P.crucifer* = 24.0–25.0 μm, see Gonzalez-Muñoz et al. 2015: fig. 3). Also, small basitrichs like those depicted in the actinopharynx of *P.crucifer* (see Gonzalez-Muñoz et al. 2015: fig. 3) were absent in *P.pinnulatus*. In tissues of *P.pulcher*, microbasic *b*-mastigophores were found (Chintiroglou and den Hartog 1995), but we did not encounter any in tissues of *P.pinnulatus*. Moreover, basitrichs in the marginal tentacles and mesenterial filaments of *P.pinnulatus* are larger than those in *P.pulcher*. The cnidom data of *P.muscosus* (shown in Carlgren 1940, 1950) largely agreed with those of *P.pinnulatus* – we hypothesize that individuals identified by Carlgren (1950) as *P.muscosus* are likely to be *P.pinnulatus* too. From all syntypes of *P.levis* examined in this study, we found microbasic *p*-mastigophores present in the tissues of marginal projection and column. This cnida was absent from the same tissues of *P.pinnulatus*. Overall, we found a difference of both cnidae type and capsule sizes among *P.pinnulatus* and its congeners. While cnidae type and size alone cannot distinguish species (Fautin 1988), when used together with other traits that are consistent (i.e., discal tentacle appearance) it appears that this feature can be useful in differentiating *Phymanthus* species.

### Morphotypes

Intraspecific morphotypes of *Phymanthus* anemones have been widely documented in primary scientific literature, with reports focused on *P.crucifer*’s variable appearance of protuberances (see Duerden 1900; González-Muñoz et al. 2015). In some individuals these are reduced knobs; in others they are entirely absent. Uncertainty persists concerning the usefulness of this character in distinguishing congeners or even members of Phymanthidae (i.e., between *Phymanthus* and *Heteranthus*; see Gonzalez-Muñoz et al. 2015). Among *P.pinnulatus* individuals collected and examined here, we encountered some specimens exhibiting this variation. Morphotypes with absent or reduced protuberances were typically encountered from the south of Singapore, although this was confined to a small number of individuals (5 out of 53) that was collected over 16 years. Like the study on *P.crucifer* by Gonzalez-Muñoz et al. (2015), we did not find an ecological cause for this. Furthermore, Gonzalez-Muñoz et al. (2015) and Brugler et al. (2018) found little genetic differentiation among *P.crucifer* morphotypes examined. While we did not have the opportunity to test for any genetic differences among *P.pinnulatus* morphotypes, we hypothesize that there is little or no variation among them. On a population basis we conclude that all morphotypes examined in this study must be of a single species, similar to observations and interpretations of Duerden (1900), Gonzalez-Muñoz et al. (2015) and Brugler et al. (2018) on *P.crucifer*. We conclude that this variation may not be extensive; overall the appearance of ramified protuberances is a useful character to distinguish members of the Phymanthidae at genus-level. Conversely, this trait is not diagnostic of *Phymanthus* species due to its variable appearance; here we infer that the discal tentacle form and cnidom are more useful and consistent for differentiating members of the genus.

### Biology

Little is known about the biology and ecology of *Phymanthus* anemones. On reproduction, Jennison (1981) found brooded juveniles within *P.crucifer*. These were encountered in individuals he had collected during the months of “December, February, and May” (Jennison 1981: 1717). In specimens of *P.pinnulatus* dissected for this study, no brooded juveniles were encountered. Most individuals studied here were collected at different times of the year, spanning more than 40 years; we hypothesize that it is unlikely that internal brooding occurs among *P.pinnulatus*. In observing individuals kept alive in the aquaria before fixation, we did not observe any evidence for asexual reproduction, as with Jennison (1981) and Lin et al. (2001).

### Morphological comparisons with *Heteranthus*

As stated on the onset, the family Phymanthidae consists of two valid and extant genera, *Phymanthus* and *Heteranthus* (Fautin 2013; 2016). Originally, members of *Heteranthus* were classified as a separate family, Heteranthidae (see Carlgren, 1900). In studying a single specimen from Vietnam (i.e., *H.insignis*), Carlgren (1943) placed both genera together in a single family, though he was not explicit on details for his rationale, remarking (Carlgren 1943: 30), “… we now know the organization of the genus better, I think that it can be brought together with *Phymanthus* in a family. Both genera are closely related to each other.” Based on his monograph published thereafter, we infer that Carlgren grouped these two genera together due to members of *Phymanthus* and *Heteranthus* having discal and marginal tentacles (Carlgren 1949). Largely, he distinguished these two genera largely on the presence of protuberances on the marginal tentacles (see Carlgren 1940: 74).

However, in his diagnosis of *Heteranthus*, Carlgren (1949: 75) further differentiates *Heteranthus* from *Phymanthus* stating that members of the former have, “… large verrucae, which at the margin are small and more numerous and overhang the fosse.” We examined the syntype of *H.verruculatus* (ZNB Cni 1852) and holotype of *H.insignis* (NRS4076) and found this character to be present: verrucae resembling conspicuous warts densely cover each marginal projection of *Heteranthus* specimens, that extend out into the fosse. These were absent on the marginal projections of all type and voucher specimens of *Phymanthus* anemones we have examined for this study (e.g. see Fig. 4F). This character clearly distinguishes the two Phymanthidae genera, despite both having discal and marginal tentacles.

## Conclusions

In Table 2, we summarise differences in discal tentacle appearance, cnidom, and type localities for eight *Phymanthus* species. These are based on details from prior publications and our own observations of type materials, if present. Of those for which we have examined type materials, eight were comprised only of one anemone species within the lot. An updated, detailed taxonomic account for the other *Phymanthus* species will be discussed in a separate manuscript.

Fautin (2013, 2016) listed eleven valid species of *Phymanthus* worldwide. Despite the comprehensive redescription of *P.pinnulatus* attempted here, taxonomic confusion still exists for nearly all remaining *Phymanthus* species. Apart from *P.crucifer*, species boundaries defining remaining members are unclear. In reviewing published descriptions of other *Phymanthus* species, apart from *P.crucifer*, we found that much of the confusion is exacerbated by a lack of thoroughness in examining type and voucher specimens. Our study addresses this limitation for *P.pinnulatus*, but other members of the genus require similar treatment along with a formal revision of the family Phymanthidae.

**Table 2.** Comparison of discal tentacle form, cnidom, and type localities of eight *Phymanthus* species. The symbol “×” indicates that the trait was found; “?” indicates that the trait was not examined in detail when the animal was first described and thereafter; a blank denotes that the trait was not observed from the species at all. Abbreviations: a, actinopharynx; c, column; pt, protuberances; dt, discal tentacles; mf, mesenterial filaments; mp, marginal projection; mt, marginal tentacles.

**Morphological traits**			***Phymanthus* species**							
			*** pinnulatus ***	*** levis ***	*** strandesi ***	*** sansibaricus ***	*** crucifer ***	*** loligo ***	*** pulcher ***	*** rhizophorae ***
**Discal tentacle form**	Papilliform				×	×	×	×	?	×
	Conspicuous			×	×	×	×	×	?	×
	Reduced		×						?	
	As a stunted tentacle			×					?	
**Cnidae**	Basitrichs	a	×	×	×	×	×	?	×	?
		c	×	×	×	×	×	?	×	?
		pt	×	×	×	×	×	?	?	?
		dt	×	×	×	×	×	?	?	?
		mf	×	×	×	×	×	?	×	?
		mp	×	×	×	×	×	?	?	?
		mt	×	×	×	×	×	?	×	?
	Microbasic *p*-masti-gophores	a	×	×	×	×	×	?		?
		c		×				?		?
		pt						?	?	?
		dt						?	?	?
		mf	×	×	×	×	×	?	×	?
		mp		×				?	?	?
		mt						?		?
	Microbasic *b*-masti-gophores	a						?		?
		c						?		?
		pt						?	?	?
		dt						?	?	?
		mf						?	×	?
		mp						?	?	?
		mt						?		?
	Spirocysts	a						?	×	?
		c						?		?
		pt	×	×	×	×	×	?		?
		dt	×	×	×	×	×	?		?
		mf						?		?
		mp						?		?
		mt	×	×	×	×	×	?	×	?
**Type locality**			Singapore	Indonesia	East Africa		Caribbean	Red Sea	Mediterranean	Indonesia
**References**			Klunzinger 1877; This study	Kwietniewski 1898	Carlgren 1900		Andrès 1883; Duerden 1900; González-Muñoz et al 2015	Andrès 1883; Carlgren 1900	Andrès 1883; Chintiroglou and den Hartog 1995	Mitchell 1890; Haddon 1898

## Supplementary Material

XML Treatment for Phymanthus pinnulatus
